# Nascent Glial Precursors in Human Bone Marrow Allow Rapid Induction of Functional Oligodendrocyte Precursors for Therapy

**DOI:** 10.3390/cells15070598

**Published:** 2026-03-27

**Authors:** Guy Lam, Kenneth Lap Kei Wu, Alex Yat Ping Tsui, Kin Wai Tam, Maximilian Tak Sui Li, Alfred Ho Lai Pao, Zora Chui-Kuen Chan, Chun Hei Kwok, Yvonne Cheuk Yin Wong, Daisy Kwok Yan Shum, Graham Ka Hon Shea, Ying Shing Chan

**Affiliations:** 1 School of Biomedical Sciences, LKS Faculty of Medicine, The University of Hong Kong, Hong Kong SAR, China; u3506833@connect.hku.hk (G.L.); alfredpao.hl@gmail.com (A.H.L.P.); shumdkhk@hku.hk (D.K.Y.S.); 2 Department of Orthopaedics and Traumatology, LKS Faculty of Medicine, The University of Hong Kong, Hong Kong SAR, China; 3 State Key Laboratory of Brain and Cognitive Sciences, The University of Hong Kong, Hong Kong SAR, China; 4 Neuroscience Research Centre, The University of Hong Kong, Hong Kong SAR, China

**Keywords:** human bone marrow stromal cells, oligodendrocyte precursor cells, remyelination

## Abstract

**Highlights:**

**What are the main findings?**

- A proliferative population of cells expressing pre-oligodendrocyte precursor cell (CD90^hi^EGFR^+^PDGFRA^+^ pre-OPC) markers was identified in human bone marrow stromal cells (hBMSCs).
- These cells can give rise to highly pure, fate-stable, and functional OPCs within 8 days in vitro.

**What is the implication of the main finding?**

- Results paint the bone marrow as a heterogeneous reservoir of progenitor cells.
- Selective enrichment and maturation of target progenitor subpopulations based on sequencing data unlocks rapid induction of desired cell types and the possibility for cell transplantation within the critical therapeutic window.

**Abstract:**

Loss of myelinating oligodendrocytes and myelin impairs motor and cognitive functions. Transplantation of autologous oligodendrocyte precursors (OPCs) holds promise for treatment of such diseases, but a protocol to derive human OPCs from a safe, ethical and accessible cell source with the rapidity required to catch the therapeutic window remains to be found. Although we previously generated myelinating glia from rat bone marrow stromal cells (BMSCs), it remains unknown if clinically sourced human BMSCs (hBMSCs) share the same potential. Moreover, whether the multipotency of BMSCs results from diverse progenitors preexisting in the bone marrow or from a single multipotent progenitor population remains unaddressed. Single-cell RNA sequencing data revealed a CD90^hi^EGFR^+^PDGFRA^+^ pre-OPC-like subpopulation within hBMSCs. With a small-molecule-based (virus-free and supporting-cell-free) two-step induction protocol designed to expand this pre-OPC population, we generated functional OPCs with high purity in eight days. These derived OPCs showed phenotypic transcriptomes and immunoprofiles. They were also capable of myelinating naked axons when transplanted into myelin-deficient shiverer mice. Results highlight how targeted enrichment and maturation of specific progenitor subpopulations within hBMSCs allows rapid induction of desired cell types. These results place hBMSCs as a robust source of OPCs, unlocking the possibility for cell transplantation therapy for myelin deficiency in the central nervous system.

## 1. Introduction

Oligodendrocytes are resident glial cells of the central nervous system [1,2] They form myelin sheaths to maintain axonal integrity, increase conduction velocity and provide metabolic support for neuronal function [3,4,5].

Despite the great complexity of the vertebrate nervous system, all neurons and most glial cells originate from the neural plate [6,7,8]. Cells in the middle of the neural plate are incorporated into the neural tube, which becomes the central nervous system (CNS) [9]. On the other hand, cells at the neural plate border then go on to become neural-crest-derived progenitors that detach from the neural tube and migrate through the embryo to generate the peripheral nervous system [10].

During neural tube development, neural stem cells within the ventricular zone give rise to neurons, followed by glial lineages, including astrocytes and oligodendrocyte precursor cells [11].

Not all neural crest stem cells differentiate into mesenchymal cells upon arriving at their destination. Small populations of neural-crest-derived stem/progenitor cells (NCSPCs) remain as progenitors even in the adult stage [12]. These have been found in many terminal migration sites, including adipose tissue [13] and bone marrow [14,15], and are proposed as a reserve for tissue regeneration and repair. NCSPCs generate not only neural tissue of the peripheral nervous system (PNS) [10,16] but also non-neural mesenchymal cell types, such as blood vessels, cartilage and bone [17]. Previously, we generated CNS oligodendrocytes from rat bone marrow stromal cells without genetic reprogramming [18]. This suggested that multipotency of the bone marrow extended even beyond peripheral tissue. Whether such diversity arises from high multipotency of NCSCs or the coexistence of multiple subpopulations of progenitors in the NCSC population remains a burning question.

Endogenous glial progenitors in the CNS proliferate and attempt to differentiate following injury [19], but this repair response is inadequate [20]. Several factors limit effective remyelination, including: (1) the hostile post-injury microenvironment characterized by inflammatory cytokines [21,22], cytotoxic mediators [23] and inhibitory molecules such as myelin-associated glycoprotein (MAG) and extracellular matrix components [24,25]; (2) insufficient numbers or recruitment of intrinsic oligodendrocyte precursor cells (OPCs) [26]; (3) the chronicity of the lesion; and (4) age-associated declines in the differentiation capacity of OPCs [27]. Collectively, these barriers mean that adult white matter struggles to achieve functionally restorative levels of remyelination following injury [28].

Naïve oligodendrocytes generated de novo exhibit substantially greater remyelination capacity than the stressed oligodendrocytes that persist after a demyelinating insult [29]. This finding is consistent with clinical and experimental evidence that in situ OPC-mediated remyelination is insufficient in chronic lesions [28] and highlights the therapeutic potential of introducing freshly derived BMSC-origin OPCs to enhance repair.

In this study, we asked if human bone marrow harbors glial stem/progenitor cells lingering from development and whether these clinically sourced cells can be used to derive myelinating glia for treatment of congenital demyelination diseases. To this end, we sequenced mRNA transcripts from bone marrow stromal cells (hBMSCs) from three healthy donors at the single-cell level. This revealed a population of cells that transcriptionally resembled the recently reported CD90^hi^EGFR^+^PDGFRA^+^ pre-oligodendrocyte precursor cells (pre-OPCs) found in the embryonic brain [30]. In the developing brain, CD90^hi^EGFR^+^PDGFRA^+^ cells mark precursors committed to oligodendroglial fate, which exclusively give rise to Olig2^+^ cells and no astrocytes [30]. We therefore hypothesized that the CD90^hi^EGFR^+^PDGFRA^+^ population found in hBMSCs might also be predisposed to an OPC cell fate. To this end, we leveraged pharmacological inhibition of BMP4 and TGFb signaling as a simple means of preventing undesirable differentiation and promoting maturation along the neuroectoderm lineage [31,32,33,34].

Here, we report a culture induction pipeline for in vitro derivation of hBMSC-derived oligodendrocyte precursors (hBMSC-OPCs). These hBMSC-OPCs were able to myelinate naked axons in vivo following intracerebral delivery into the corpus callosum (CC) of myelin-deficient shiverer mice. Results thus give promise to the sourcing of adult bone marrow for neural progenitors that can be expanded and differentiated into OPCs and tapped for remyelination therapy.

## 2. Materials and Methods

### 2.1. Human Bone Marrow Sample Collection and Culture Expansion

Human bone marrow samples were obtained from the femur of three healthy donors aged 33–45 years with written consent according to protocol UW21-442, approved by the Institutional Review Board of the University of Hong Kong/Hospital Authority Hong Kong West Cluster (HKU/HA HKW IRB).

Bone marrow samples were diluted in sterile phosphate buffered saline (PBS). Cells were pelleted by centrifugation (450× *g*, 5 min, 4 °C) and then resuspended in a growth medium comprising MEM-alpha medium (Thermo Fisher Scientific, Waltham, MA, USA) containing 10% FBS (Biosera, Kansas City, MO, USA) and allowed to adhere to the culture vessel for 48 h at 37 °C with 5% CO_2_. Gentle washes with PBS and a medium change after 48 h removed suspended cells. hBMSCs were passaged into fresh growth medium when they reached 90% confluence by detaching with TrypLE Express (Thermo Fisher Scientific, Waltham, MA, USA).

All experiments were conducted with hBMSC cultures between passages 3 and 6. hBMSCs could be cryopreserved in liquid nitrogen by resuspending in cryopreservation medium containing growth medium supplemented with 10% DMSO (Sigma-Aldrich, St Louis, MO, USA) with no observable impact on viability or yield of OPCs.

### 2.2. Shiverer Mouse Model

To demonstrate in vivo myelination capacity of hBMSC-derived OPCs, these cells were transplanted to the corpus callosum (CC) of homozygous shiverer mice (C3Fe.SWV-MBPShi/J, The Jackson Laboratory, Bar Harbor, ME, USA) that naturally lack myelin due to knockout of the myelin basic protein (MBP) gene. hBMSC-OPCs were transplanted into the right corpus callosum of postnatal day 7 shiverer mice (bregma −1 mm, medial–lateral 1 mm, dorsal–ventral 1.5 mm) by stereotaxic injection using a Neuros syringe (Hamilton, Reno, NV, USA), based on established protocols [1]. An amount of 1 μL of either hBMSC-OPCs or hBMSCs (both at 1 × 10^6^ cells/μL in PBS) was administered into the brain of each mouse. The number of mice that were transplanted with hBMSC-OPCs derived from each donor and their grouping into 12 and 16 weeks post-transplantation groups are listed in Table S1. Controls were transplanted with hBMSCs (n = 2 mice). Mice were provided with food and water ad libitum under a 12 h light–dark cycle. Mice were randomly assigned into OPC or control group, but order of treatments was not controlled. All transplanted animals were used for g-ratio analysis. Parallel treatments for OPCs derived from 3 patients were technically unfeasible due to availability of shiverer mice.

Taking advantage of the tolerogenic period in neonates [35], immunosuppression was not required. Experimental protocols were approved by the Committee for Use of Live Animals in Teaching and Research, The University of Hong Kong (approval number 4704-18).

### 2.3. Induction of Glial Progenitors

To start OPC induction, hBMSCs were seeded onto Matrigel (Corning, Corning, NY, USA)-coated 10 cm plastic culture dishes (Techno Plastic Products AG, Trasadingen, Switzerland) at a density of 1 × 10^5^ cells per dish. DMEM/F12 (Thermo Fisher Scientific, Waltham, MA, USA) was supplemented with 1% N2, 2% B27 (Thermo Fisher Scientific, Waltham, MA, USA), 1 mM sodium pyruvate, 60 µg/mL N-acetylcysteine, 100 nM LDN 193189 and 10 nM SB-431542. Cultures were exposed to LDN 193189 and SB-431542 for 72 h. The medium is known as glial-inducing medium (GIM).

### 2.4. Maturation of Glial Progenitors Along the OL Lineage

To obtain OPCs, hBMSCs were passaged onto poly-D-lysine and laminin-coated culture dishes using Accutase after 72 h in LDN 193189- and SB-431542-containing medium. The medium was changed to OPC commitment medium (OCM), devoid of the small molecule inhibitors mentioned above. The medium comprises: advanced DMEM/F12 (Thermo Fisher Scientific, Waltham, MA, USA) supplemented with 0.5% N_2_, 0.25% Glutamax, 1 mM sodium pyruvate, 60 µg/mL N-acetylcysteine, 20 ng/mL PDGF-AA, and 20 ng/mL bFGF, and optionally to increase cell yield, FBS (1%). OPCs were culture-expanded in OCM and passaged once after 3 days to obtain sufficient cells for in vivo transplantation. Early-stage OPCs are optimal for in vivo transplantation with respect to their proliferative and migration capacity. PDGF-AA and bFGF increase OPC proliferation and maintain OPC identity yet block oligodendrocyte differentiation and prevent terminal fate commitment toward mature OLs. OPCs could then be dissociated using Accutase for transplantation or cryopreserved in OPC cryopreservation medium comprising 90% GIM (DMEM/F12 medium supplemented with N2, bFGF, PDGF-AA) with 10% DMSO in liquid nitrogen if desired. To generate carboxyfluorescein succinimidyl ester (CFSE)-marked hBMSC-OPCs (Figure S2c), hBMSC-OPCs were first washed with 1xPBS and incubated with CFSE for 10 min at 37 °C. After 2 further washes with 1xPBS, hBMSC-OPCs were dissociated from the dish using Accutase and collected in an Eppendorf tube. Cells were pelleted with brief centrifugation (250× *g*, 3 min) and washed twice with ice-cold PBS to remove Accutase. Cells were kept in ice-cold PBS and transplanted within 20 min.

### 2.5. Multiplexed Single-Cell RNA Sequencing and Bioinformatics Analysis

Transcriptomes of naïve hBMSCs were acquired from all three patient samples at the single-cell level to elucidate the heterogeneity of progenitors in human bone marrow and to evaluate the degree of inter-patient variability of progenitor pool composition. Single-cell transcriptomes from hBMSCs of each donor were also acquired after 72 h exposure to LDN 193189- and SB-431542, as well as at the end of the OPC induction process after 8 days of induction and fate commitment.

Briefly, 1 × 10^6^ cells at the desired stage were detached with TrypLE or Accutase and resuspended in ice-cold PBS for multiplexing. Cell viability was above 90% for all samples as determined by Trypan Blue exclusion. Cells from each donor at each stage along the induction process were labeled with a unique set of Cell Multiplexing Oligos using a Single Cell Gene Expression v3.1 (Dual Index) kit (CD000391, 10X Genomics, Pleasanton, CA, USA). After labeling and washing, an equal number of cells from each patient at the same stage in the induction process were mixed for encapsulation, Gel Bead-in Emulsion (GEM) generation and library preparation according to the manufacturer’s protocol (Chromium controller, 10X Genomics, Genomics, Pleasanton, CA, USA). The resulting libraries were sequenced to a depth of 450–550 million reads per library (Illumina NextSeq 2000 System, San Diego, CA, USA). Cell counts per donor, genes per cell, cells per gene and percentage mitochondrial reads are shown in Figure S1d,e. De-multiplexing of sequencing data was accomplished using Cell Ranger multi-software.

Downstream single-cell sequence analysis was accomplished using Scanpy (version 1.8.1). Low-quality cells with less than 100 genes detected and genes detected in less than 3 cells were removed from analysis. Gene expression values were normalized to counts per million and log-transformed for comparison across samples. Dimension reduction was performed using the top 2048 high-variable genes identified by Scanpy with the “seurat-v3” algorithm. Batch correction was achieved using the iterative self-learning algorithm DESC (version 2.1.1). For visualization of cellular heterogeneity, tSNE plots were generated using Scanpy. Clusters were assigned by Leiden clustering at a resolution of 0.25 (all data) or 0.5 (fine clustering of hBMSCs). CD90^hi^ was defined as having THY1 expression above the non-zero mean of THY1 within the hBMSC population. Trajectory analysis was conducted using the PAGA pipeline [36] following a standardized procedure. This comprehensive methodological approach allowed us to unveil single-cell gene expression patterns and provided insights into the dynamic processes associated with glial differentiation pathways.

ScFates was also used to perform pseudotime linear (curve) trajectory inference from single-cell data. Raw data and demultiplexed matrix files can be downloaded from NCBI GEO GSE302455.

### 2.6. Flow Cytometry

For fluorescence-assisted sorting of CD90^hi^ EGFR^+^PDGFRA^+^ cells from hBMSCs, anti-EGFR-FITC, anti-CD90-PE/Cy7 and anti-PDGFRA-biotin antibodies [30] were diluted 1:1000 in ice-cold 3% BSA in PBS. Live hBMSCs were first blocked with ice-cold 3% BSA in PBS and then incubated with a cocktail of antibodies for 30 min on ice. After three rounds of washing with ice-cold PBS, hBMSCs were incubated with streptavidin-APC diluted 1:1000 in ice-cold 3% BSA in PBS for 30 min on ice. After a final three rounds of washing with ice-cold PBS, triple-positive cells were sorted out using a BD Influx Cell Sorter with a 100 μm nozzle. Unstained and singly stained cells were used to set the background and compensation settings. Sorted hBMSCs were cultured in GIM.

For analysis of marker expression at various stages during the protocol, cell suspensions were fixed in 4% PFA for 5 min and then sedimented by centrifugation (250× *g*, 5 min). Cell pellets were gently washed with PBS and then resuspended in a blocking buffer comprising PBS with 3% *v*/*v* NGS and 0.1% *v*/*v* Triton X100 for 30 min. The cells were incubated with selected primary antibodies (listed in Table S2) for 2 h at 4 °C followed by the appropriate Alexa Fluor^®^ secondary antibodies (diluted 1:500 in blocking buffer, Life Technologies, Carlsbad, CA, USA) for 30 min. Immunolabeled cells were subjected to flow cytometric analysis with the use of a NovoCyte Advanteon BVR analyzer. A minimum of 10,000 cells were analyzed per experiment. Primary antibody was omitted to determine the background level of fluorescence in control experiments.

### 2.7. Immunofluorescence of Cultured Cells

Cultured cells were fixed with 4% PFA and washed 3 times with PBS before incubation in blocking buffer comprising PBS with 3% w/v bovine serum albumin (Sigma-Aldrich, St. Louis, MO, United States) and 0.1% *v*/*v* Triton X100 (Sigma-Aldrich, St. Louis, MO, United States) for 30 min. The appropriate primary antibodies were diluted in blocking buffer and incubated with samples at 4 °C overnight (listed in Table S2). Immunoreactivity was visualized using the appropriate Alexa Fluor^®^ secondary antibodies (diluted 1:500 in blocking buffer, Thermo Fisher Scientific, Waltham, MA, USA) for 60 min. After 3 rounds of washing in PBS, Hoechst 33258 (Sigma-Aldrich) was used to counterstain nuclei. Images were viewed under an Olympus IX71 inverted fluorescence microscope equipped with an Olympus DP71 camera for image capture. For cell counting, 10 random fields were captured and a minimum of 300 cells were counted per experiment by investigators blinded to the grouping.

Mouse and rabbit isotype controls (1:200, Thermo Fisher Scientific, Waltham, MA, USA) were used in control experiments.

### 2.8. Sample Preparation and Immunohistochemistry

At 12 and 16 weeks post-injection, mice were euthanized by overdose of pentobarbital (150 mg/kg i.p., Alfasan, Woerden, The Netherlands) followed by exsanguination via transcardial perfusion with ice-cold saline followed by 4% PFA. Brain tissue was dissected, post-fixed overnight in 4% PFA at 4 °C, and cryoprotected in 30% sucrose solution. Coronal cryosections (50 μm) containing the corpus callosum were prepared (NX50 cryostat, Thermo Fischer Scientific, Waltham, MA, USA). Tissue sections were permeabilized and blocked with 1% Triton X-100 and 3% BSA in PBS for 60 min and then incubated with primary antibodies overnight at 4 °C (listed in Table S2) followed by the appropriate fluorophore-conjugated Alexa Fluor^®^ secondary antibodies (diluted 1:500 in blocking buffer, Thermo Fisher Scientific, Waltham, MA, USA) overnight at 4 °C. Cell nuclei were counterstained with Hoechst 33258. The stained sections were rinsed in PBS and mounted for viewing and image capture under confocal microscopy (Zeiss LSM 800, München, Germany).

### 2.9. Transmission Electron Microscopy

Corpora callosa from PFA-fixed brains of cell-transplanted shiverer mice were cut into 1 mm (anterior–posterior) × 1 mm (dorsal–ventral) × 3 mm (mediolateral) pieces. These corpus callosum-containing samples were further fixed with 1% osmium tetroxide and stained with 1% uranyl acetate before re-embedding in Epon. Sagittal sections (75–90 nm thick) of the corpus callosum were picked up on formvar/carbon-coated 75 mesh Cu grids and stained for 20 s in 1:1 super-saturated uranyl acetate in acetone followed by 0.2% lead citrate. Grids were viewed under a Philips CM100 transmission electron microscope (Eindhoven, The Netherlands). Percentage myelination was calculated by dividing the number of myelinated axons by the total number of identified axons. More than 120 axons were counted for each donor source (number of mice listed in Table S1). Axons were identified based on stereotypical ultrastructure, including lack of nucleus or rough endoplasmic reticulum and high density of microtubules. Only axons with a diameter between 150 nm and 1 mm were included for percentage myelination calculation, as axons beyond these dimensions could not be identified confidently. Compact myelination was identified by characteristic major dense line structure. Only axons that had more than 2 full layers of compact myelin were counted as myelinated. Analysis of myelin morphology was accomplished by calculating the myelin g-ratio within imaged areas of 100 μm^2^ of the corpus callosum.

### 2.10. Statistical Analysis

Researchers conducting sectioning, staining, image acquisition and flow cytometry analysis were blinded to the experimental groups. Blinding was technically infeasible for single-cell transcriptome preparation since the researchers had to remain cognizant of the sample identity to ensure data integrity.

Cell enrichment was evaluated by Fisher’s exact test in Scipy. Benjamini–Hochberg correction was used to control false discovery rate for multiple testing. An FDR < 0.05 was considered significant. Enrichment of gene ontology (GO) terms for Biological Processes was evaluated using the python package GSEApy (version 1.1.12) with the cutoff set at *p* = 0.05. *p*-values for significant enrichment of selected GO terms were plotted for each of the cell stages as −log_10_ (adjusted *p*-value). Statistical analyses of g-ratios were performed using GraphPad Prism Software 10 (GraphPad Software Inc., San Diego, CA, USA). Data are presented as mean ± S.D. Shapiro–Wilk, D’Agostino and Pearson tests were used to confirm normality, with equal variance pre-tested with Levene’s test. One-way ANOVA with post hoc Tukey’s test was used to compare g-ratios. *p*-values < 0.05 were considered significant.

## 3. Results

### 3.1. Characterization of Human BMSCs

**Figure 1:**
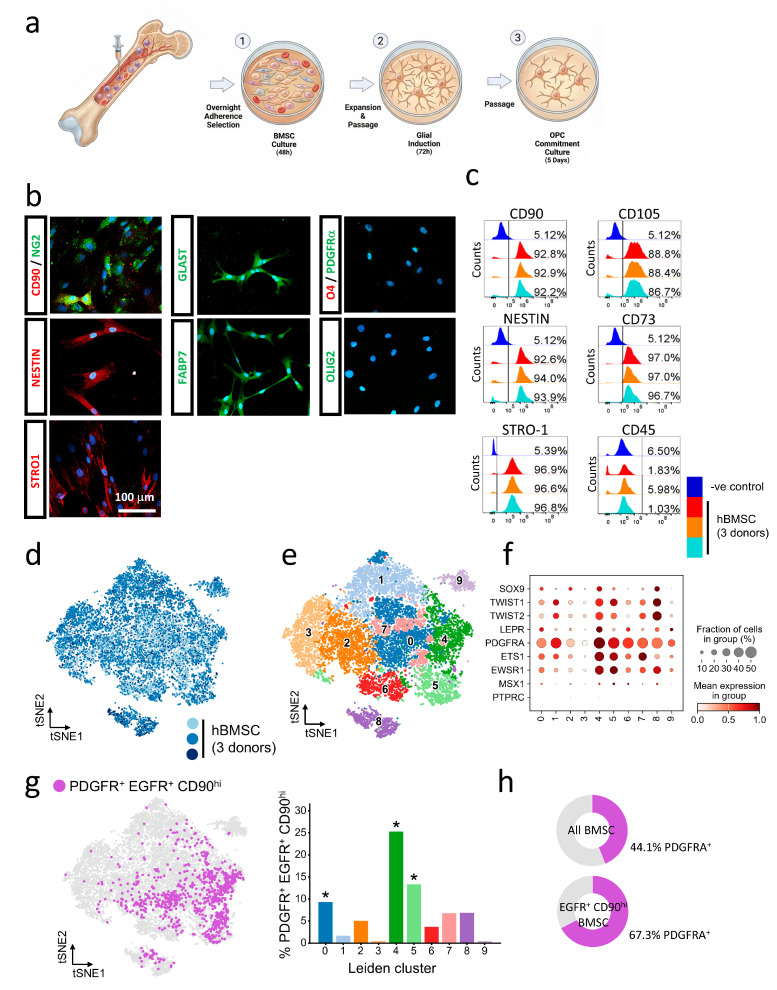
Immunophenotyping of hBMSCs and discovery of a pre-OPC like population. (**a**) Human BMSCs (1) were isolated from bone marrow aspirates and purified by brief adherent culture. This was followed by induction to hBMSC-GPC (2) by 3-day exposure to GIM. Resultant hBMSC-GPCs were differentiated into hBMSC-OPCs (3) by 5-day treatment with OCM. (**b**) hBMSCs expressed the mesenchymal stem/stromal cell (MSC) markers CD90 (Thy1) and STRO-1, the neural progenitor/stem markers NESTIN, the radial glial cell/astrocyte marker GLAST and the radial glial cell marker FABP7. Oligodendrocyte lineage markers O4, PDGFRA and Olig2 were not detectable in hBMSCs. (**c**) Marker expression was consistent across cells obtained from 3 donors. Flow cytometry analysis showed high expression for MSC markers: CD90^+^ (MSC marker) 92.63 ± 0.31%, CD105^+^ (MSC marker) 87.97 ± 0.91%, CD73^+^ (MSC marker) 96.9 ± 0.14%, STRO-1+(MSC marker) 96.77 ± 0.12%, NESTIN^+^ (neural stem marker) 93.5 ± 0.64%, CD45^+^ (hematopoietic marker, negative selection marker for MSCs and NSCs) 2.95 ± 2.17%. Mean ± SD. Blue: isotypic primary antibody negative control. Red: donor 11 hBMSC. Orange: donor 15 hBMSC. Turquoise: donor 16 hBMSC. (**d**) tSNE plot of hBMSC transcriptomes colored by donor identity. Homogenous mixing of cells from 3 donors suggests that hBMSCs from individuals have similar transcriptomes. (**e**) Leiden clustering of hBMSC transcriptomes revealed a total of 10 transcriptionally distinct subpopulations within the hBMSC pool. (**f**) Dotplot showing expression of canonical mesenchymal stromal cell markers including LEPR, PDGFRA and post-migratory NCSC markers including SOX9 and TWIST1 in clusters 0, 4 and 5. (**g**) Putative pre-OPCs with characteristic PDGFRA^+^ EGFR^+^ CD90^hi^ gene expression within hBMSCs are highlighted in purple. PDGFRA^+^ EGFR^+^ CD90^hi^ cells are significantly enriched in clusters 0, 4, 5 and 7. * FDR < 0.05. (**h**) PDGFRA expression is significantly enriched in EGFR^+^ CD90^hi+^ hBMSCs compared to the whole hBMSC population (*p* = 4.55 × 10^−12^).

Human BMSCs obtained from the femur of three healthy adult donors were expanded separately in adherent culture (Figure 1a). Three rounds of passaging were conducted to remove any residual CD45-expressing hematopoietic stem cells and CD31-expressing endothelial/angiogenic cells (Figure S1a). hBMSCs were positive for a panel of mesenchymal stem cell markers CD90, CD73, CD105, and STRO-1 (Figure 1b,c) but remained negative for OPC markers including O4, Olig2, and PDGFRA (Figure 1b). Additionally, hBMSCs expressed multiple progenitor markers Nestin and NG2 (Figure 1b). Quantitation of key markers across hBMSCs from all three donors by flow cytometry confirmed that the immunophenotypic profile was consistent across all three hBMSC samples (Figure 1c). Results showed that in vitro expansion of hBMSCs was effective in removing hematopoietic stem cells but retaining mesenchymal/neural progenitor cell populations.

### 3.2. Discovery of a Developing CNS Glial Progenitor Population in hBMSCs

BMSCs can give rise to diverse neural and non-neural tissues. We previously derived functional Schwann cells and oligodendrocyte precursors from rat BMSCs [18,37]. Furthermore, derivation of astrocytes and neurons from bone marrow has also been reported [38]. Given the diverse cell types capable of being derived from bone marrow that arise from multiple germ layers, we reasoned that it was unlikely that a single pool of multipotent stem cells could evade maturation in the human bone marrow. Instead, we hypothesized that multiple predefined pools of progenitors biased towards distinct lineages could coexist in the bone marrow, amenable to selective activation and maturation by induction and derivation protocols.

To answer this question, we profiled the transcriptome of hBMSCs at single-cell resolution. Transcriptomes of BMSCs from three donors were intermingled when plotted as a tSNE plot (Figure 1d). This suggested that the transcriptional signatures of hBMSCs were highly similar between individuals, corroborating our flow cytometry immunophenotyping results. Natural clustering revealed a total of 10 transcriptionally distinct subpopulations within the hBMSC pool (Figure 1e), supporting the hypothesis that multiple progenitor subpopulations coexist in the bone marrow. Canonical mesenchymal stromal cell markers LEPR^hi^/PDGFRA^hi^/PTPRC^low^ [12] were enriched in clusters 0, 4, and 5 (Figure 1f), which also expressed the post-migratory neural crest (NCSC) markers SOX9 and TWIST1 [14]. Further, we identified a subpopulation of cells with the characteristic CD90^hi^ EGFR^+^PDGFRA^+^ triple-expressing profile of pre-OPCs in the mid-gestation human brain (Figure 1f) [30]. Such cells accounted for 8.2% of all hBMSCs (Figure 1f) and were significantly enriched within clusters 0, 4 and 5 (Figure 1g, adjusted *p* = 2.94 × 10^−3^, 2.42 × 10^−97^, 5.71 × 10^−10^, respectively). Notably, 67% of cells within the EGFR^+^ CD90^hi^ population were PDGFRA^+^ compared to only 44% PDGFRA^+^ across all hBMSCs (Figure 1h, *p* = 4.55 × 10^−12^). This suggested that neural progenitors within the hBMSCs significantly encompassed these putative glial progenitors.

Given such enrichment of cells with a pre-OPC-like transcriptomic signature within this putative neural progenitor subpopulation in the bone marrow, we asked if we could directly promote glial progenitor cell fate commitment [39] from hBMSCs. Given developmental proximity to the neural crest observed in the hBMSC marker profile [12,13,14,40], with naturally occurring low levels of glial markers [12,13,14], we reasoned that BMP inhibition would disrupt maintenance of cell stemness [41], allowing these pre-OPC-like subpopulations of hBMSCs to respond to further glial induction cues in the medium (such as growth factors like heregulin, bFGF, or PDGF) that promote fate commitment.

We applied dual SMAD inhibitors LDN-193189 and SB431542, inhibiting SMAD1/2/3/5/8 phosphorylation [42] in the glial-inducing medium (GIM) for hBMSCs in adherent culture to inhibit BMP4 and TGFb signaling (Figure 1a, step 2).

**Figure 2:**
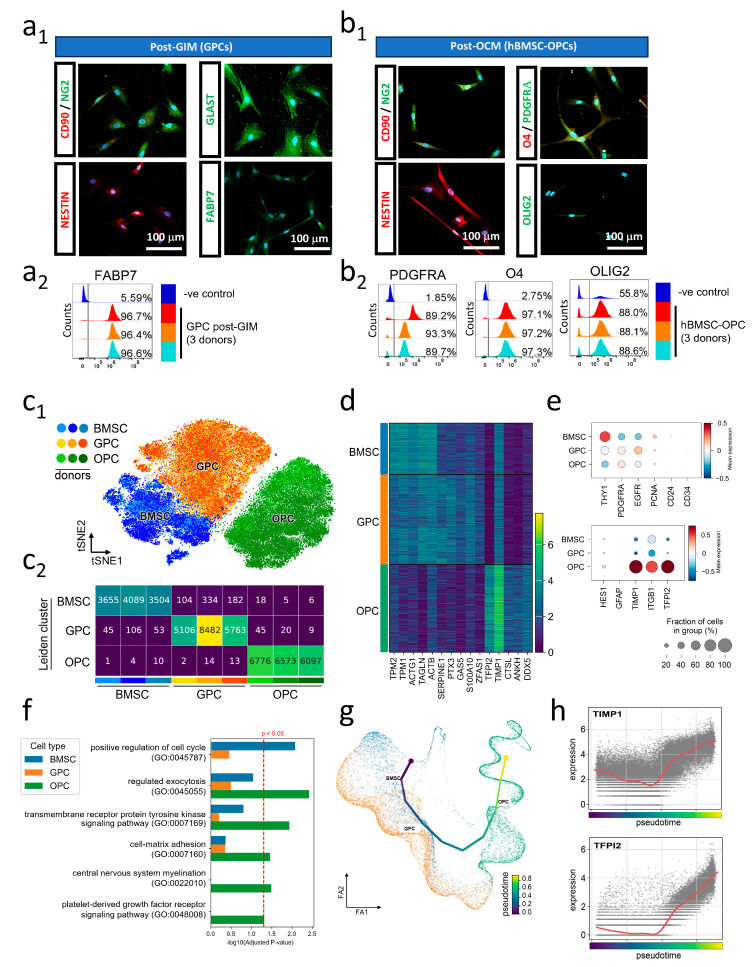
Immunophenotyping and transcriptomic analysis of hBMSC maturation to OPC through GPC induction. (**a1**) Immunophenotyping of GPCs after 3 days of GIM treatment. Expression of progenitor markers GLAST, FABP7, NESTIN, NG2 and CD90 was unaffected. (**a2**) A total of 96.57 ± 0.12% of hBMSC-GPCs were FABP7^+^ (radial glia and neural stem cell marker) after 3 days of GIM treatment. (**b1**) Immunophenotyping of hBMSC-OPCs. Canonical OPC markers Olig2, O4 and PDGFRA were expressed after 5 days of OCM treatment. NESTIN, NG2 and CD90 expression was retained. (**b2**) Eight days after start of induction, the majority of hBMSC-OPCs expressed PDGFRA (early-stage OPC marker) 90.73 ± 1.83%, O4 (mid-to-late O4) 97.2 ± 0.08%, and Olig2 (all oligodendrocyte lineage cells) 88.23 ± 0.26%. (**c1**) t-SNE-plot of hBMSC (blues), hBMSC-GPCs (oranges) and hBMSC-OPCs (greens). Cells from 3 donors were well mixed at all stages, demonstrating reproducibility across samples, while the 3 cell types (hBMSCs, hBMSC-GPCs, hBMSC-OPCs) were distinctly clustered. (**c2**) Number of cells from each sequencing sample clustered into each of the 3 main Leiden clusters corresponding to BMSC, GPC and OPC. Majority of cells are correctly clustered into the expected cluster. (**d**) Heatmap of top 6 differentially expressed genes in hBMSC (blue), dual SMAD inhibitor-treated hBMSC-GPCs (orange) and hBMSC-OPCs (green). (**e**) Dotplot highlighting expression changes of key marker genes across the 3 cell stages. PDGFRA, EGFR, TIMP1 and ITGB1 are enriched across induction. HES1 expression increases as cells mature into hBMSC-OPCs, whereas THY1 (CD90) expression decreases. (**f**) Selected GO Biological Process terms that are significantly enriched in cell types. (**g**) Pseudotime trajectory of hBMSCs, hBMSC-GPCs and hBMSC-OPCs. (**h**) Expression of TIMP1 and TFPI2 along pseudotime trajectory.

After 3 days of incubation in the inhibitor-containing GIM, the hBMSCs assumed a more elongated phenotype, while expression of glial progenitor markers FABP7 and GLAST (Figure 2(a1), right panel), mesenchymal stem cell marker CD90 and progenitor markers Nestin and NG2 were retained (Figure 2(a1), left panel). Flow cytometry confirmed that 3-day treatment with LDN-193189 and SB431542, inhibitors of BMP4 and TGFb signaling, resulted in the expression of FABP7 in over 96% of cells across three samples tested (Figure 2(a2)). This is consistent with a glial progenitor identity and suggests that glial progenitors in hBMSCs retain their potential to progress along a glial maturation trajectory.

### 3.3. Establishment of OP Cell Fate

The culture medium was changed to OCM containing PDGF-AA (Figure 1a) 3 days post-glial induction. bFGF was also included to promote growth of the pre-OPC population to obtain sufficient cell number for transplant. Inclusion of FBS in the culture medium increased the rate of cell growth but was not an absolute requirement for obtaining OPCs (Figure S1a–c), providing a serum-free alternative for future clinical applications. After 5 days, these putative OPCs became 80% confluent and were resuspended for transplant. Immunophenotyping of the resulting cells revealed robust expression of the OPC markers PDGFRA, O4 and Olig2 (Figure 2(b1)). This is in line with flow cytometry results that indicated that at least 88% of cells from all three donors were positive for PDGFRA, O4 and Olig2 (Figure 2(b2)). Our single-cell transcriptomic characterization reflected a gradual increase in PDGFRA expression (canonical OPC marker) across induction and maturation (Figure 2e, top panel), accompanied by a gradual reduction in expression of CD90 (THY1). These results thus demonstrated that pre-existing glial progenitors in hBMSC were able to mature into OPCs within 8 days. This is also reinforced by progressive upregulation of HES1 mRNA from BMSC to OPC cultures accompanied by low GFAP expression throughout, further supporting that inhibition of TGFb signaling successfully prevented stimulation of maturation along the astrocyte lineage [42] (Figure 2e, lower panel).

To reinforce our hypothesis that the CD90^hi^ EGFR^+^PDGFRA^+^ triple-positive subpopulation within the hBMSC pool was preferentially expanded and matured into OPCs by our protocol, we sorted out this population by fluorescence-assisted cell sorting and cultured the triple-positive and non-triple-positive cells normally in GIM followed by OCM (Figure S2a). Notably, non-triple-positive hBMSCs had poor survival in the protocol, while the sorted triple-positive population adopted typical OPC morphology after 5 days in OCM (Figure S2b). This suggested that survival and expansion of these putative neural crest glial progenitors were indeed favored.

### 3.4. In Vivo Maturation of OPCs into Myelinating Oligodendrocytes After Transplant into Myelin-Deficient Shiverer Mice

**Figure 3:**
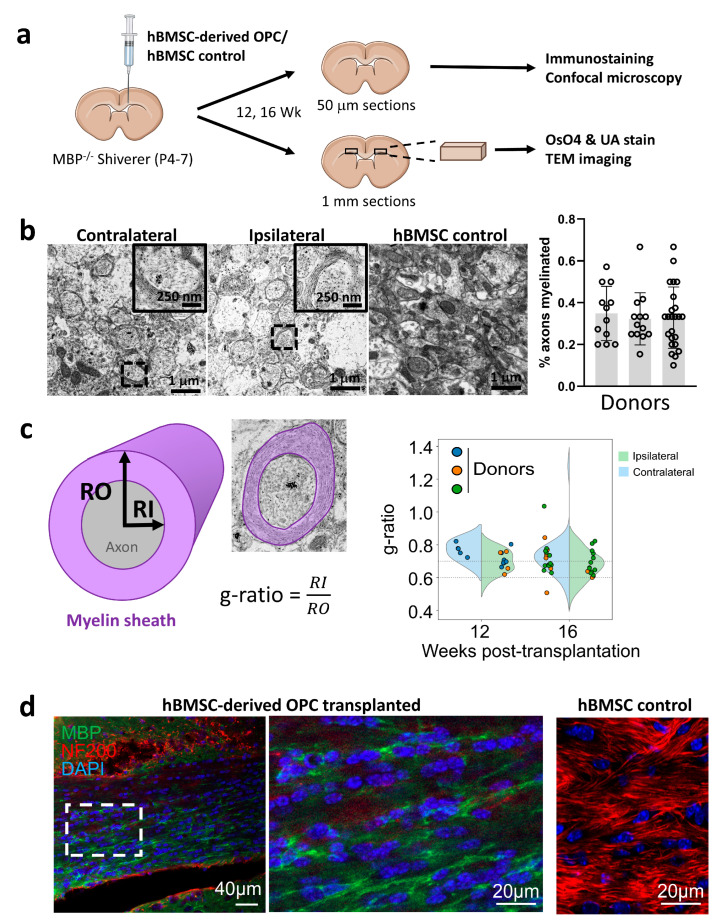
Derived OPCs migrated, matured and myelinated corpus callosum axons with compact myelin in myelin-deficient mice. (**a**) Schematic of in vivo myelination assay. hBMSC-OPCs were transplanted into the corpus callosum of the right hemisphere. The animals were harvested at 12 and 16 weeks post-injection. Myelination was detected by immunohistochemistry/confocal microscopy and osmium tetroxide-UA staining/transmission electron microscopy (TEM). (**b**) **Left**: Sagittal sections of the brain under electron microscopy. Compact myelin rings were identified at both the contralateral and ipsilateral side of the corpus callosum in derived OPC treatment group at week 12. Myelination is absent in hBMSC-injected control shiverer mice. **Right**: Graph showing the percentage myelination achieved after transplantation of hBMSC-OPCs derived from each of the three hBMSC samples 12 weeks post-surgery. Each dot represents the percentage myelination (quantified per field of view of 30 μm^2^) in one mouse. Bars represent mean ± SD. (**c**) G-ratio (RI/RO) quantification schematic of myelinated axons, obtained by dividing inner diameter (RI) by the overall diameter (RO) of the axon. G-ratio of myelinated axons at 12 weeks was 0.71 ± 0.07 and that at 16 weeks was 0.70 ± 0.10. The g-ratio on the contralateral side (0.73 ± 0.11, left) was slightly higher than that of the ipsilateral side (0.69 ± 0.08, right) but was not statistically significant. Dots represent g-ratio of each mouse and are colored according to the source of hBMSCs used to generate the hBMSC-OPCs in each mouse. Dotted horizontal lines indicate the range of g-ratio in wildtype mice. (**d**) Immunohistochemistry (IHC) of tissue sections post-hBMSC-derived OPC transplantation demonstrated MBP positivity extending along the fiber tracts marked by NF200-positivity. MBP staining was not observed in control shiverer mice injected with hBMSCs.

We next tested if OPCs prepared according to our protocol are capable of myelinating naked axons. To this end, we transplanted them into the corpus callosum of shiverer mice that have a deletion mutation that largely removes the myelin basic protein (MBP) gene, making them incapable of forming compact myelin [43] (Figure 3a). Control mice were transplanted with hBMSCs. Transplanted mice were sacrificed 12 weeks post-transplantation for analysis of myelin formation. Analysis of TEM images revealed formation of myelin around axon bundles in the corpus callosum of mice transplanted with hBMSC-derived OPCs (Figure 3b) but not in controls that received hBMSC transplant. This was congruent with the existence of MBP-positive cells displaying typical oligodendrocyte morphology with multiple processes extending along axons (labeled with NF200) in the corpus callosum of mice transplanted with hBMSC-derived OPCs (Figure 3d).

Notably, although hBMSC-derived OPCs were only injected into the right-hand side corpus callosum, myelin formation could also be detected on the contralateral side (Figure 3b). This indicated that hBMSC-derived OPCs were capable of migration and targeting toward naked axons, similar to endogenous OPCs in the brain. To further confirm the origin of myelinating cells in the corpus callosum, carboxyfluorescein succinimidyl ester (CFSE)-labeled hBMSC-OPCs were injected in a limited number of shiverer mice. At 12 weeks post-injection, cells displaying typical oligodendrocyte morphology and immunoreactivity for MBP in the corpus callosum were exclusively CFSE-positive (Figure S2c). An average myelination ratio of 27.72 ± 8.17% was achieved in transplanted corpus callosum (N = 3 donors), contrasted with no observable compact myelin in hBMSC-transplanted controls. G-ratio of quantified myelin sheaths across 12- (0.71 ± 0.07) and 16-week (0.70 ± 0.10) timepoints are similar. G-ratio of contralateral corpus callosum sections across both timepoints is 0.73 ± 0.11 (0.76 ± 0.06, 0.73 ± 0.06, 0.73 ± 0.13 for each donor), while g-ratio of ipsilateral corpus callosum sections across both timepoints is 0.69 ± 0.08 (0.70 ± 0.06, 0.66 ± 0.07, 0.69 ± 0.08 for each donor). While thinner myelination is demonstrated on the ipsilateral side of the corpus callosum contralateral to the injection site, migration and myelination is demonstrated (difference was not statistically significant) (Figure 3c). No evidence of tumorigenesis or astrocytosis was observed in mice engrafted with hBMSC-OPCs up to 5 months.

### 3.5. Inferring the OPC Induction Mechanism from hBMSCs Using Single-Cell Transcriptomics

To explore possible mechanisms underlying rapid generation of hBMSC-derived OPCs, hBMSCs treated with BMP4 and TGFb signaling inhibitors (3 days GIM) (5212, 8830 and 5958 cells from each donor, respectively), and hBMSC-OPCs (6839, 6598 and 6112 hBMSC-OPCs from each donor, respectively) were transcriptomically profiled along with hBMSCs (3701, 4199 and 3567 hBMSCs from each donor, respectively) at the single-cell level (Figure S1d). Unsupervised clustering of single cells on a t-SNE plot resulted in cells clustering according to cell type (Figure 2(c1)). This was verified statistically by examining the cell composition of Leiden clusters (Figure 2(c2)). Good mixing of cells from all three patients within each individual cell type not only showed that hBMSCs obtained were reproducible across patients but also demonstrated robustness of the induction protocol at all stages. Detection of transcripts across all three patients was similar, with a statistically insignificant reduction in the number of genes expressed as hBMSCs progressed along maturation programs towards OPCs (Figure S1d). Cells at the three stages of OPC derivation had distinct marker genes (Figure 2d). Notably, expression of hBMSC marker genes persisted in the GPC stage but declined upon further maturation into OPCs (Figure 2d).

To better understand the maturation process, we examined the enriched GO terms for Biological Process (Figure 2f). Positive regulation of cell cycle was significantly enriched in hBMSCs but dropped sharply throughout maturation, reflecting reduced proliferation and self-renewal as progenitors become fate-committed. Cell matrix adhesion and central nervous system myelination became significantly enriched in hBMSC-OPCs, suggesting that hBMSC-OPCs were poised to myelinate axons on the transcriptional level. Genes involved in transmembrane receptor protein tyrosine kinase signaling were significantly enriched in hBMSC-OPCs (Figure 2f).

Pseudotime analysis on combined BMSC, GPC and OPC stage transcriptomes from three patients revealed an aligned trajectory, which was indicative of progressive cell differentiation from multipotency to the glial terminal state (Figure 2g). Annotation of sample cohort identity on the trajectory revealed a clear shift of cell identity from BMSC to GPC and then OPC along the progenitor (BMSC), bridge (GPC) and progeny (OPC) stages (Figure 2g). This was accompanied by a gradual increase in expression of the canonical OPC marker PDGFRA, as well as a gradual reduction in expression of the negative regulator of OPC maturation CD90 (THY1) along the trajectory (Figure 2e). Expression profiles of these canonical genes supported the validity of the assembled trajectory.

We wondered if non-canonical pathways that promote OPC maturation could be discovered from transcriptomics data. Close examination of the data revealed interesting changes in expression of genes that were suggested to participate in OPC maturation (Figure 2e). For example, expression of the positive regulator of OPC maturation, tissue inhibitor of metalloproteinase 1 (TIMP1), increased gradually as cells progressed through the induction process. TIMP-1 knockout mice exhibit delayed CNS myelination during development and impaired remyelination following injury [44], and TIMP-1 was reported to drive OPC maturation [45,46], while that of tissue factor pathway inhibitor (TFPI2), known to promote Olig2 in zebrafish, was increased only at the final OP induction stage (Figure 2h). Notably, CD63 and ITGB1, the components of the signaling receptor complex which mediates the metalloproteinase-independent activity of TIMP1 [46], were detectable in nearly all cells throughout the OPC induction process (Figure 2e). These analyses suggest that in addition to established FGF and PDGF-AA signaling supplied in OPC induction medium, our induction protocol further promoted upregulation of autologous TIMP1 signaling through CD63 and β1-integrin to drive β-catenin-mediated OPC maturation [46]. Further gene perturbation studies will be required to prove that these pathways predicted based on transcriptomic changes do indeed drive the production of OPCs from hBMSCs.

## 4. Discussion

The current study describes a novel, rapid protocol to generate functional human OPs from hBMSCs. Both female (39 years old) and male (33 and 45 years old) BMSC samples used in this study: (1) did not show significant differences in transcriptomes, and (2) gave high-purity OPCs with comparable yield by the induction protocol. We hypothesize that our protocol harnessed progenitors in the human bone marrow with a transcriptional signature similar to glial progenitor cells in the developing brain. This not only shortens the time required to obtain functional OPCs with high purity but also yields a simplified protocol that only involves adherent culture without the need for an intermediate suspension culture stage to form neurospheres.

In our study, we observed compact myelin formation (Figure 3b,d) on both the contralateral and ipsilateral sides of the cell-injected brain, with multilamellar sheaths and a clear major dense line when viewed under TEM. This demonstrated the migration and myelinating capacity of our hBMSC-derived OPCs. The g-ratio is a biophysically meaningful metric that links structure (myelin vs. axon size) to function (conduction efficiency) [47]. The similar g-ratios of the quantified myelin sheaths at 12 and 16 weeks indicated integration of transplanted OPCs and the stability of myelination 3 months post-transplantation. The g-ratio of remyelinated axons in shiverer mice was not different from that reported for wildtype adult mice, 0.72 ± 0.04 [48,49]. OPCs derived from each of the three hBMSC samples produced similar g-ratios in shiverer mice. The similarity in myelin formation was in line with the similarity of transcriptomes between the three samples used. Further to the g-ratio, the nodal organization and ultrastructure was also close to normal developmental myelination [4], though with slightly weaker radial components and less adaxonal myelin [4,50]. Lack of a significant difference in OPC density between the ipsilateral and contralateral sides suggested that hBMSC-OPCs retained high migration capacity.

We transplanted cells into immune-competent shiverer mice within the neonatal immune tolerogenic period, during which the developing immune system can develop lifelong tolerance to nonself antigens [35]. This approach circumvents the need for strict asepsis in immune-compromised mice or undue stress resulting from daily injection of immune-suppressive drugs to the neonates and has been used in similar studies to demonstrate the myelination potential of OPCs [50,51]. Notably, if tolerance was not established, immune response or inflammation in the mouse would favor the elimination of the transplanted cells [21,22]. As such, successful remyelination in an immune-hostile environment would further highlight the robustness of the derived cells. Resilience in an inflammatory environment is desirable since both acute lesions and white matter disease are often accompanied by chronic inflammation and immune cell influx to the myelin.

The putative glial progenitor population of CD90^hi^ EGFR^+^ PDGFRA^+^ triple-positive cells harbored within the hBMSCs was hypothesized to originate from neural crest stem cells that migrated into the bone marrow during development [14]. Post-migratory neural crest (NCSC) markers SOX9 and TWIST1 were found co-expressing and enriched within the population. While one cannot entirely rule out the possibility that such marker expression and differentiation potential resulted from coincidence, or in vitro culture conditions, this instead suggests a neural crest lineage. Given that a definitive demonstration of neural crest stem and progenitor cell migration into the bone marrow during development using transgenic lineage tracing is unfeasible for humans, transcriptomic similarity nonetheless provided data in support of this hypothesis.

This study provides proof-of-principle data supporting the efficacy of hBMSC-OPCs in remyelination therapy. Results prove that the derivation of pure, functional cultures of hOPCs can be achieved within a clinically relevant timeline and with repeatability. Given the prospects of such findings for cell transplantation therapy, we sought to test whether xeno-free induction of OPCs was possible. As a proof-of-principle test, we omitted the only component in the protocol for which a GMP version did not exist—bovine serum used in OPC induction medium. This had no observable impact on the purity or function of OPCs obtained, but the growth rate of these hBMSC-derived OPCs was lower than those grown in OP induction medium containing 1% FBS (Figure S1). Nonetheless, to yield a cell product that is fully qualified for cell therapy, complete adoption of xeno-free PIC/S-GMP-grade products would be required.

However, several hurdles remain to be addressed before adoption of such cells in therapy can be considered. First, with neonatal establishment of immune tolerance, the microenvironment within shiverer brains is pro-myelinating due to the lack of compact myelin along with the associated growth-inhibitory molecules on mature myelin (e.g., Nogo-A, MAG, OMgp), as well as a lack of physical competition from existing myelin on axons. Moreover, reactive astrocytes and microglia in the chronic dysmyelination microenvironment could secrete factors promoting OPC proliferation and survival as part of a compensatory or repair mechanism in the shiverer mouse. As such, the stability of fate commitment and the myelination capacity of OPCs transplanted into the shiverer mouse brain might be enhanced when compared to less permissive situations, such as transplantation into traumatic models or demyelination or degenerative situations with residual myelin and myelin debris. Second, the post-transplantation follow-up duration in this study was limited to 5 months by the short lifespan of shiverer mice. While we observed no evidence of tumorigenesis or astrocytosis in engrafted mice up to 5 months, further long-term trials are needed to definitively prove the long-term safety of such cells. Third, the number of hBMSC samples used in this study was limited. As such, a rigorous analysis of the effect of sex and age could not be conducted. Finally, one order of magnitude increase in cells, from 10^6^ used in this study to 10^7^ used in clinical trials, would be needed for therapeutic purposes [52]. Nonetheless, due to the simplicity of the protocol and the fact that the derivation efficiency was not impacted by cryopreservation of hBMSCs, it is conceivable that a large-scale preparation of hBMSC-OPCs either with a single source of hBMSCs to achieve clinically relevant cell numbers or with the use of multiple hBMSC samples to validate the efficacy of this protocol over a more diverse range of ages, could be easily implemented.

This protocol stands out from previous attempts to derive OPCs from other sources by the short induction time required to achieve high-purity OPCs with in vivo myelinogenic capacity. Analysis of single-cell transcriptomes gave several indications of the underlying reason. Firstly, TIMP1, a key temporal regulator of myelin formation [53], was upregulated over the course of OPC induction. Secondly, the receptors for TIMP1, CD63 and β1-integrin [46] were persistently expressed in a high percentage of cells at all stages. Upregulation of TIMP1 therefore allows cell-autonomous activation of Akt (protein kinase B)-β-catenin signaling via the CD63/β1-integrin receptor complex to promote specification and maturation of glial progenitors into OPCs [44].

Single-cell transcriptomics further revealed upregulation of the serine protease inhibitor TFPI2 in the final OPC induction stage. TIMP1 and TGF-β2 signaling are positive regulators of OPC maturation (Figure 2h, upper panel). TFPI2 (Figure 2h, lower panel) is known to enhance TGFβ2 signaling by disrupting the interaction between SMURF2 and SMAD7, thereby preventing SMURF2-mediated inhibition of the TGFβ receptor complex and increasing cellular responsiveness to TGFβ [54]. While inhibition of TGFβ signaling was required for selective expansion of glial precursors in the DS stage, subsequent re-activation of the TGFβ2 pathway is necessary for OPCs to exit the cell cycle and undergo terminal differentiation into mature, myelinating oligodendrocytes [41]. Enhanced upregulation of TFPI2 could therefore mediate the transition of glial progenitors in the DS stage to mature OPCs that are able to respond to TGFβ signaling in the CNS with myelination.

Serendipitous triggering of endogenous pathways for OPC maturation may underlie this alternative protocol for yielding highly pure OPCs from small numbers of CD90^hi^EGFR^+^PDGFRA^+^ glial progenitors recoverable from hBMSCs. While this hypothesis remains to be proven definitively, OPCs produced following the protocol were nonetheless functional, capable of homing to denuded CNS axons and myelinating such axons in the shiverer mouse model.

## 5. Conclusions

Our findings support the hypothesis that the bone marrow harbors diverse cell types, specifically those descending from the neural crest lineage [12,13,14]. We used single-cell transcriptomes to reveal heterogeneous subpopulations within the BMSC pool, which encompass post-migratory neural crest stem cell (NCSC) that are SOX9^+^TWIST1^+^ and within these cell, a CD90^hi^EGFR^+^PDGFRA^+^ pre-OPC subpopulation that showed specific progenitor transcriptional signatures. Our protocol stands out by the short induction time required to achieve high-purity OPCs with in vivo myelinogenic capacity.

While our findings do not disprove the existence of multipotent populations among BMSCs [55], we reason that rational design of culture protocols that target pre-existing progenitors in the hBMSC pool allows rapid derivation of desired target cell types while at the same time minimizing the risk of undesirable lineage variability.

Overall, our study uncovers the heterogeneity of progenitor subpopulations within the bone marrow and suggests a route towards rapid generation of OPCs that could rescue CNS myelin deficiency from autoimmune, degenerative and traumatic conditions.

## 6. Patents

G.L, K.L.K.W and A.Y.P.T are co-inventors on the invention patent filed relating to the work in this manuscript.

## Data Availability

The data presented in this study are openly available in NCBI GEO at GSE302455.

## Supplementary Materials

The following supporting information can be downloaded at: https://www.mdpi.com/article/10.3390/cells15070598/s1, Supplementary Figure S1. (a) ICC staining showing adherence-selective culture devoid of CD45+ hematopoietic stem and CD31+ angiogenic cells across all three stages: (1) hBMSC, (3) Post-OCM and optionally, Post-OCM, without FBS. Scale bar: 100 µm. (b) Phase contrast images of (1) hBMSC, (2) Post-GIM, (3) Post-OCM and optionally, Post-OCM, without FBS. Scale bar: 200 µm. (c) CD90, NG2, O4, PDGFRa, Nestin and Olig2 are expressed in post-OCM OPCs; cells devoid of Stro1 expression. Scale bar: 100 µm. (d) Violin plots showing genes by counts, total counts, and percentage mitochondrial genes across BMSC, GPCs and OPCs. (e) Total number of qualified BMSC, GPCs and OPCs that were sequenced from each of the 3 donor samples. Supplementary Figure S2. (a) Schematic showing the workflow to FACS-sort PDGFR^+^ EGFR^+^ CD90^hi^ population in hBMSCs to fate-commit to OPCs. Triple-positive and non-triple-positive cells from the same sort were then cultured in induction (GIM) (72 h) and matured in fate-commitment media (OCM) (5 days), in parallel. (b) Phase contrast images of PDGFR^+^ EGFR^+^ CD90^hi^ cells 24 h post-sorting and 5 days in OCM, scale bar: 1000 µm. Transition of bipolar GPCs to round morphology was seen in triple-positive population after 5 days, which reflected gradual fate commitment. Extensive cell death only seen across OCM maturation in non-triple-positive cells. Insets show magnified view of cells. (c) Confocal image of CFSE (green)-labeled hBMSC-OPCs in corpus callosum of shiverer mice 12 weeks after transplantation. Labeled cells expressed MBP (yellow) and extended multiple processes along the axons marked by NF200 (red). White arrows point to CFSE-containing vesicles in MBP+ cells. Individual channels displayed on the right. Supplementary Figure S3. Single-channel b&w images corresponding to merged immunostaining images shown in Figure 1b. Supplementary Figure S4. (a) Single channel b&w images corresponding to merged immunostaining images shown in Figure 2(a1). (b) Single channel b&w images corresponding to merged immunostaining images shown in Figure 2(b1). Table S1. Summary of mice per group for hBMSC-OPC transplant; Table S2. List of antibodies used for immunofluorescence staining and flow cytometry.

## Abbreviations

The following abbreviations are used in this manuscript:

BMP4	Bone Morphogenetic Protein 4
BMSCs	Bone marrow stromal cells
CFSE	Carboxyfluorescein succinimidyl ester
CNS	Central nervous system
CC	Corpus callosum
GIM	Glial-inducing medium
MAG	Myelin-associated glycoprotein
NCSPC	Neural-crest-derived stem/progenitor cells
OLs	Oligodendrocytes
OPCs	Oligodendrocyte precursors
OCM	OPC commitment media
Pre-OPCs	Pre-oligodendrocyte precursors cells
PBS	Phosphate buffered saline
PNS	Peripheral nervous system
TEM	Transmission electron microscopy
TGF-β	Transforming Growth Factor Beta
