# Supplementary material for: Nascent Glial Precursors in Human Bone Marrow Allow Rapid Induction of Functional Oligodendrocyte Precursors for Therapy
Source: Cells. 2026 Mar 27;15(7):598. doi: 10.3390/cells15070598 (PMC13072390; doi:10.3390/cells15070598)
Supplement: Supplementary file 1 [file cells-15-00598-s001.zip › cells-4053555-supplementary figures.pdf]

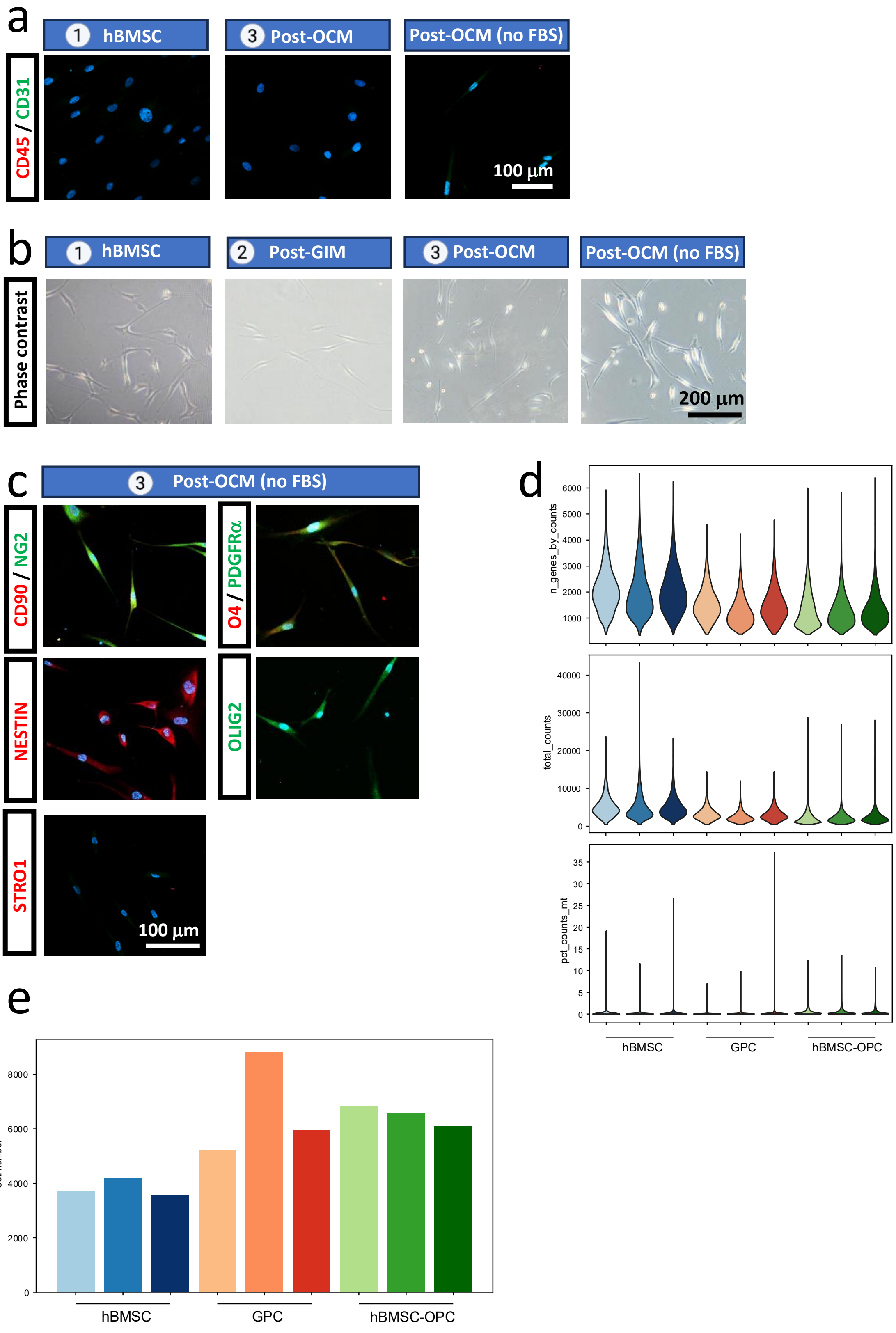

Supplementary Fig S1. (a) ICC staining showing adherence-selective culture devoid of CD45+ hematopoietic stem and CD31+ angiogenic cells across all three stages: 1) hBMSC, 3) Post-OCM and optionally, Post-OCM, without FBS. Scale bar: 100μm. (b) Phase contrast images of 1) hBMSC, 2) Post-GIM, 3) Post-OCM and optionally, Post-OCM, without FBS. Scale bar: 200μm. (c) CD90, NG2, O4, PDGFRa, Nestin and Olig2 are expressed in Post-OCM OPCs; cells devoid of Stro1 expression. Scale bar: 100μm. (d) Violin plots showing genes by counts, total counts, and percentage mitochondrial genes across BMSC, GPCs and OPCs. (e) Total number of qualified BMSC, GPCs and OPCs that were sequenced from each of the 3 donor samples.

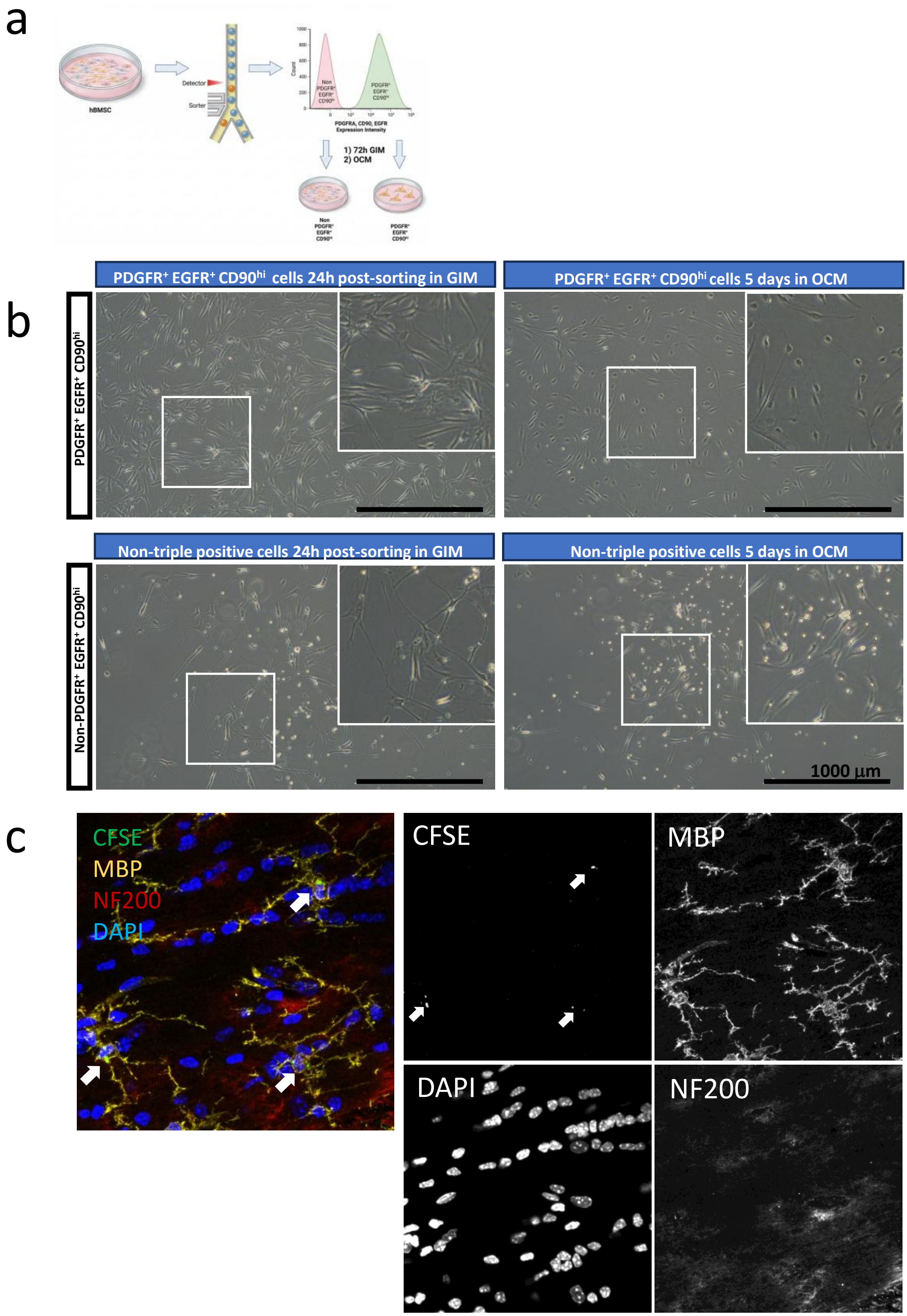

Supplementary Fig S2. (a) Schematic showing the workflow to FACS-sort PDGFR^{+} EGFR^{+} CD90^{hi} population in hBMSCs to fate commit to OPCs. Triple positive and non-triple positive cells from the same sort were then cultured in induction (GIM)(72 hours) and matured in fate commitment media (OCM) (5 days), in parallel. (b) Phase contrast images of PDGFR^{+} EGFR^{+} CD90^{hi} cells 24h post-sorting and 5 days in OCM, Scale bar: 1000µm. Transition of bipolar GPCs to round morphology was seen in triple positive population after 5 days, which reflected gradual fate-commitment. Extensive cell death only seen across OCM maturation in non-triple positive cells. Insets show magnified view of cells. (c) Confocal image of CFSE (green)-labelled hBMSC-OPCs in corpus callosum of shiverer mice 12 weeks after transplantation. Labelled cells expressed MBP (yellow) and extended multiple processes along the axons marked by NF200 (red). White arrows point to CFSE-containing vesicles in MBP^{+} cells. Individual channels displayed on the right.

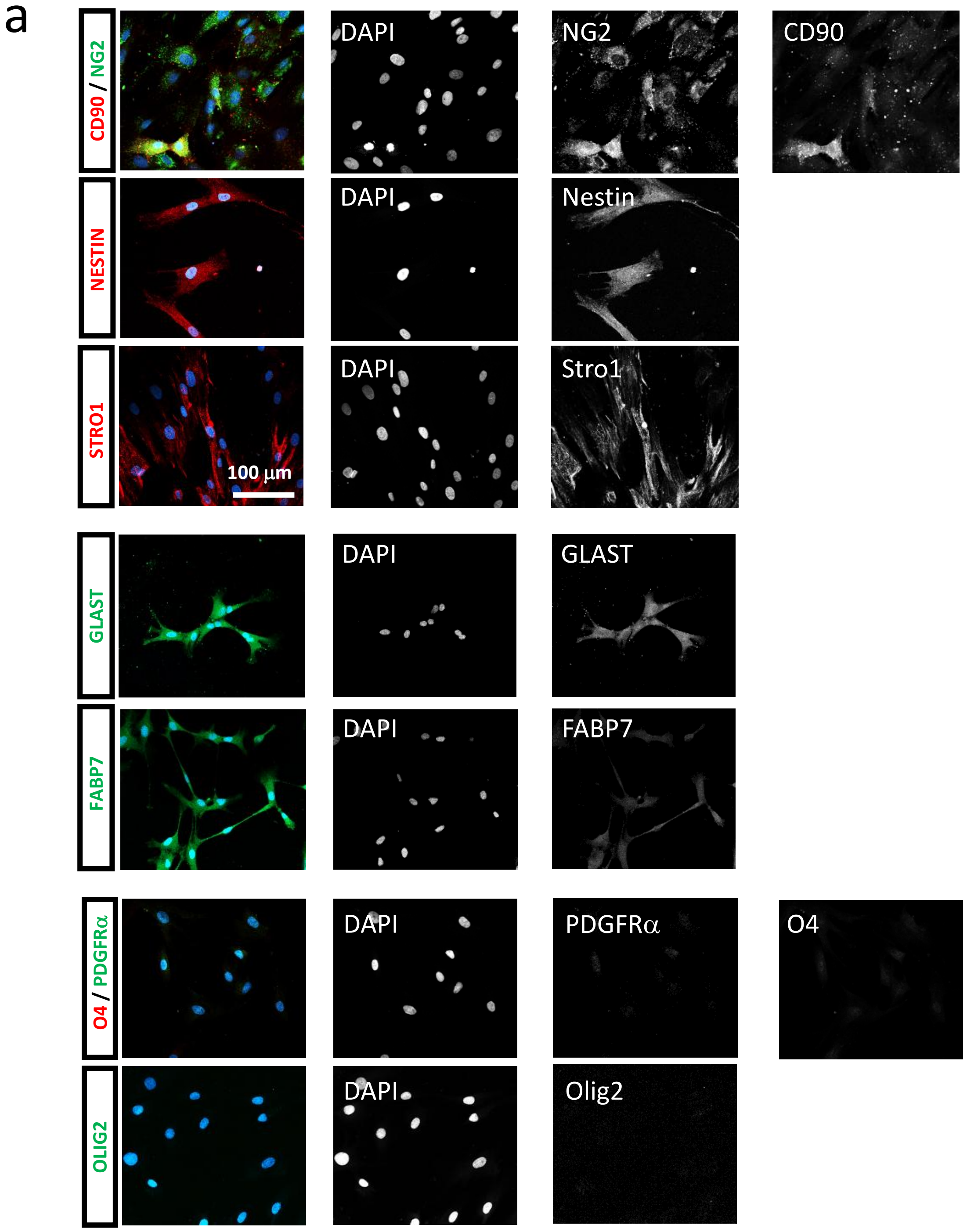

Supplementary Fig S3. Single channel monochrome images for corresponding to merged immunostaining images shown in Fig. 1b.

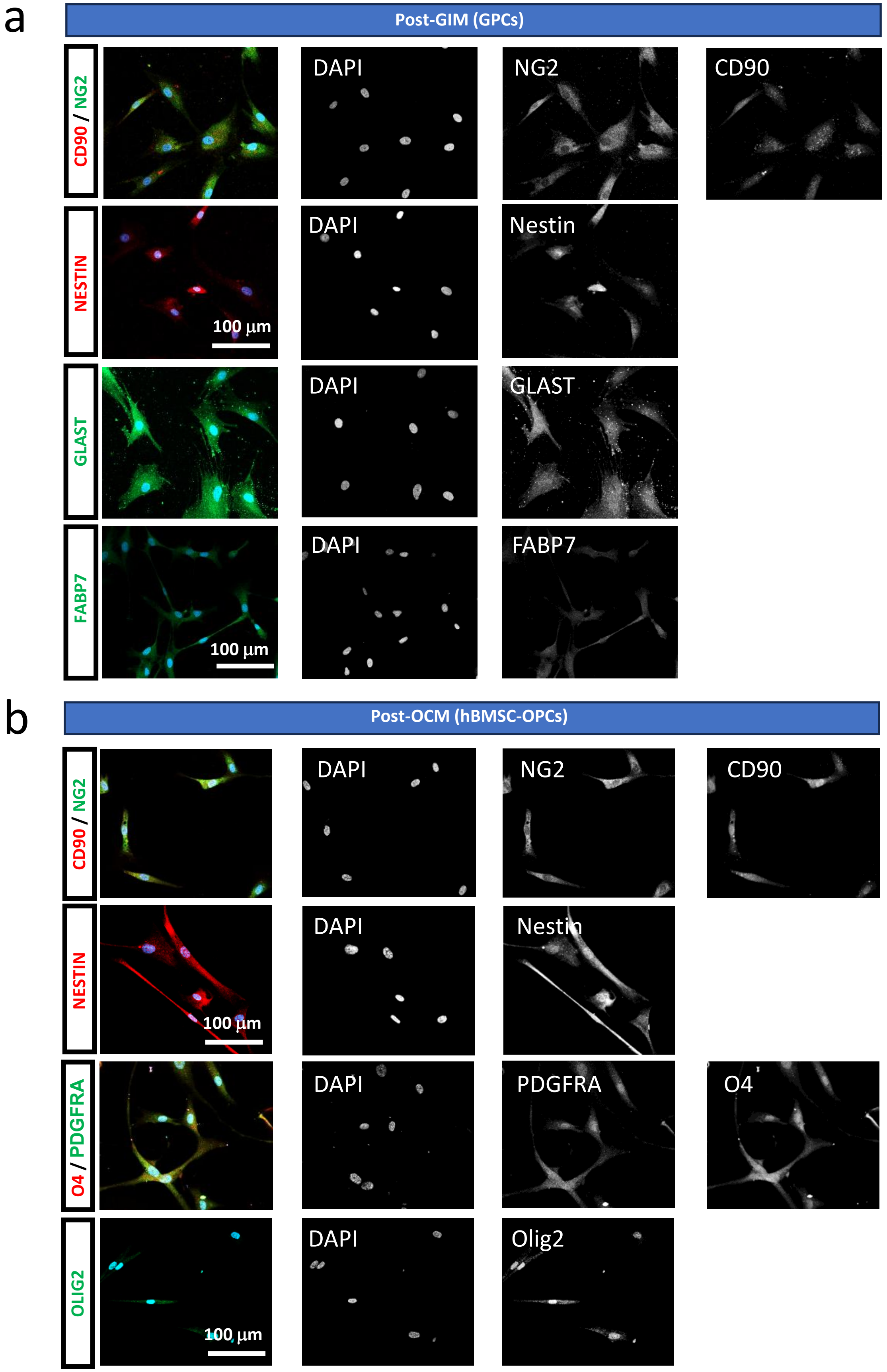

Supplementary Fig S4. (a) Single channel b&w images for corresponding to merged immunostaining images shown in Fig. 2a1. b) Single channel b&w images for corresponding to merged immunostaining images shown in Fig. 2b1.
