# Supplementary material for: Nascent Glial Precursors in Human Bone Marrow Allow Rapid Induction of Functional Oligodendrocyte Precursors for Therapy
Source: Cells. 2026 Mar 27;15(7):598. doi: 10.3390/cells15070598 (PMC13072390; doi:10.3390/cells15070598)
Supplement: Supplementary file 1 [file cells-15-00598-s001.zip › Table S2 Antibody sources.pdf]

Table S2. List of antibodies used for immunofluorescence staining and flow cytometry

Immunofluorescence staining				
**Antigen**	**Host**	**Catalogue number**	**Company**	**Dilution**
CD31	Rabbit	Nb100-2284	Abcam	1:100
CD45	Mouse	05-1410	Sigma-Aldrich	1:100
CD90	Mouse	555593	BD Pharmingen	1:100
FABP7	Rabbit	PA5-24949	Invitrogen	1:100
GLAST	Rabbit	Ab416	Abcam	1:10
HES1	Rabbit	PCRP-Hes1-1A3	DSHB	1:100
Nestin	Mouse	MAB1259	R&D Systems	1:100
NG2	Mouse	14-6504-82	Invitrogen	1:100
O4	Mouse	MAB1326	R&D Systems	1:400
Olig2	Rabbit	AB9610	Sigma-Aldrich	1:500
PAX6	Mouse	PAX6	DSHB	1:100
PDGFRa	Rabbit	PA5-16571	Invitrogen	1:500
SOX2	Mouse	MAB2018	R&D Systems	1:50
STRO1	Mouse IgM	MAB1038	R&D Systems	1:50
MBP	Mouse	BCN8.1.1E11	DSHB	1:200
NF200	Rabbit	N4142	Sigma-Aldrich	1:200
Donkey anti-Mouse IgG, Alexa Fluor 488	Donkey	A21202	Invitrogen	1:500
Donkey anti-Rabbit IgG, Alexa Fluor 488	Donkey	A21206	Invitrogen	1:500
Goat anti-Mouse IgG, Alexa Fluor 594	Goat	A11005	Invitrogen	1:500
Goat anti-Mouse IgM, Alexa Fluor 594	Goat	A21044	Invitrogen	1:500
Goat anti-Rabbit IgG, Alexa Fluor 594	Goat	A11037	Invitrogen	1:500

Flow cytometry				
**Antigen**	**Host**	**Catalogue number**	**Company**	**Dilution**
CD45	Mouse	05-1410	Sigma-Aldrich	1:100
CD73	Mouse	550256	BD Pharmingen	1:50
CD90	Mouse	555593	BD Pharmingen	1:100
CD105	Mouse	05-1424	Sigma-Aldrich	1:100
FABP7	Rabbit	PA5-24949	Invitrogen	1:100
Nestin	Mouse	MAB1259	R&D Systems	1:100
O4	Mouse	MAB1326	R&D Systems	1:400
Olig2	Rabbit	AB9610	Sigma-Aldrich	1:500
PDGFRa	Rabbit	PA5-16571	Invitrogen	1:500
STRO1	Mouse IgM	MAB1038	R&D Systems	1:50
Donkey anti-Mouse IgG, Alexa Fluor 488	Donkey	A21202	Invitrogen	1:500
Donkey anti-Rabbit IgG, Alexa Fluor 488	Donkey	A21206	Invitrogen	1:500
Goat anti-Mouse IgM, Alexa Fluor 594	Goat	A21044	Invitrogen	1:500
